# *ORAI1* Genetic Polymorphisms Associated with the Susceptibility of Atopic Dermatitis in Japanese and Taiwanese Populations

**DOI:** 10.1371/journal.pone.0029387

**Published:** 2012-01-13

**Authors:** Wei-Chiao Chang, Chih-Hung Lee, Tomomitsu Hirota, Li-Fang Wang, Satoru Doi, Akihiko Miyatake, Tadao Enomoto, Kaori Tomita, Masafumi Sakashita, Takechiyo Yamada, Shigeharu Fujieda, Koji Ebe, Hidehisa Saeki, Satoshi Takeuchi, Masutaka Furue, Wei-Chiao Chen, Yi-Ching Chiu, Wei Pin Chang, Chien-Hui Hong, Edward Hsi, Suh-Hang Hank Juo, Hsin-Su Yu, Yusuke Nakamura, Mayumi Tamari

**Affiliations:** 1 Laboratory for Respiratory Diseases, Center for Genomic Medicine, The Institute of Physical and Chemical Research (RIKEN), Kanagawa, Japan; 2 Department of Pediatric Allergy, Osaka Prefectural Medical Center for Respiratory and Allergic Diseases, Osaka, Japan; 3 Miyatake Asthma Clinic, Osaka, Japan; 4 NPO Japan Health Promotion Supporting Network, Wakayama, Japan; 5 Division of Otorhinolaryngology Head and Neck Surgery, University of Fukui, Fukui, Japan; 6 Takao Hospital, Kyoto, Japan; 7 Department of Dermatology, The Jikei University School of Medicine, Tokyo, Japan; 8 Department of Dermatology, Graduate School of Medical Sciences, Kyushu University, Fukuoka, Japan; 9 Laboratory of Molecular Medicine, The Institute of Medical Science, The University of Tokyo, Tokyo, Japan; 10 Department of Medical Genetics, College of Medicine, Kaohsiung Medical University, Kaohsiung, Taiwan; 11 Department of Healthcare Management, Yuanpei University, HsinChu, Taiwan; 12 Cancer Center, Kaohsiung Medical University Hospital, Kaohsiung, Taiwan; 13 Department of Dermatology, Graduate Institute of Medicine, Kaohsiung Medical University, Kaohsiung, Taiwan; 14 Department of Dermatology, National Taiwan University College of Medicine, Taipei, Taiwan; 15 Center for Resources, Research, and Development, Kaohsiung Medical University, Kaohsiung, Taiwan; 16 Department of Medical Research, Kaohsiung Medical University Hospital, Kaohsiung, Taiwan; Ohio State University Medical Center, United States of America

## Abstract

Atopic dermatitis is a chronic inflammatory skin disease. Multiple genetic and environmental factors are thought to be responsible for susceptibility to AD. In this study, we collected 2,478 DNA samples including 209 AD patients and 729 control subjects from Taiwanese population and 513 AD patients and 1027 control subject from Japanese population for sequencing and genotyping *ORAI1*. A total of 14 genetic variants including 3 novel single-nucleotide polymorphisms (SNPs) in the *ORAI1* gene were identified. Our results indicated that a non-synonymous SNP (rs3741596, Ser218Gly) associated with the susceptibility of AD in the Japanese population but not in the Taiwanese population. However, there is another SNP of *ORAI1* (rs3741595) associated with the risk of AD in the Taiwanese population but not in the Japanese population. Taken together, our results indicated that genetic polymorphisms of *ORAI1* are very likely to be involved in the susceptibility of AD.

## Introduction

Atopic dermatitis (AD) or childhood eczema is a chronic relapsing inflammatory skin disease [1] that usually associated with a family history of atopic disorders such as allergic rhinitis and bronchial asthma [2], [3], [4]. There has been a dramatic increase in the prevalence of AD in the last decade. Although the pathogenesis of AD remains elusive, multiple genetic and environmental factors are thought to contribute to the disease onset [2], [3], [4]. Genes associated with skin-barrier formation and adaptive immunity have been implicated in the development of AD. For example, filaggrin (FLG) is essential for the maintenance of the skin-barrier function. Genetic mutations in FLG are significantly associated with the risk of AD and elevated immunoglobulin E (IgE) levels [5]. In addition, single nucleotide polymorphisms (SNPs) in Toll-like receptors (TLRs), ST2, IL-3, IL-4, IL-5, IL12RB1, and IL-13 have been shown to be associated with the pathogenesis of AD [6], [7], [8], [9], [10], [11], [12]. The results from a genome-wide association study (GWAS) have indicated the complex involvement of multiple loci in the susceptibility of human AD [13].

Despite all the knowledge, the treatment of severe AD remains a challenge. Clinical trials have indicated that cyclosporine is an effective treatment option in children with AD. Short-term treatment with cyclosporine has shown to alleviate disease activity [14]. Cyclosporine, an immunosuppressant, functions as a phosphatase inhibitor that prevents the translocation of calcium-dependent transcription factor—nuclear factor of activated T cells (NFAT). The influx of calcium through store-operated calcium (SOC) channels is one of the major pathways to increase the intracellular calcium concentration in non-excitable cells such as the mast cells and T lymphocytes [15]. In mast cells, short-term SOC influx has been shown to result in the secretion of inflammatory molecules such as arachidonic acids and leukotriene C_4_ [16], [17]. In T lymphocytes, the nuclear translocation of NFAT could be driven by the SOC-mediated calcineurin signaling pathway in order to control immune responses [18]. The molecular components of the SOC channels were first identified in patients with severe combined immune deficiency (SCID) syndrome [19]. *ORAI1* gen encoding Orai1 protein is one of the major proteins of SOC channels. A point mutation created in the *ORAI1* gene resulted in the reduction of calcium influx through the SOC channels and dysfunction of the immune system. *ORAI1*-knockout mice exhibited defective mast cells and attenuated cytokine release [20].

In our study, we first conducted LD (linkage disequilibrium) mapping of the *ORAI1* gene, performed a case-control association study and showed a haplotype analysis. We then tested the correlation between the *ORAI1* genetic polymorphisms and the expression level of the *ORAI1* transcript. Our results support a functional role of *ORAI1* polymorphisms in the susceptibility of human AD in both Japanese and Taiwanese population.

## Materials and Methods

### Subjects

A total of 513 atopic dermatitis patients were recruited in Takao Hospital and the University of Tokyo. All subjects with atopic dermatitis were diagnosed according to the criteria of Hannifin and Rajka [21]. There are four major criteria, including pruritus, chronic or relapsing dermatitis, dermatitis affecting flexural surfaces in adults, and a personal or family history of cutaneous or respiratory atopy. There are 23 minor criteria, such as hypopigmented patch, infraorbital darkening, cheilitis, hyperlinerized palm, and elevated IgE, etc. To be included, the patients had to meet three major criteria plus at least one minor criterion or two major criteria plus at least three minor criteria. Japanese subjects with AD were recruited from several hospitals and diagnosed according to the criteria of Hanifin and Rajka by dermatologists. A total of 839 adult control individuals who had no history of bronchial asthma, allergic rhinitis and atopic dermatitis were recruited by detailed doctors' interviews. A total of 188 healthy individuals who had no history of atopic dermatitis based on a questionnaire were recruited in Fukui University (3).

Information of these participants is provided in Table S1. All individuals were unrelated Japanese and gave written informed consent to participate in the study according to the rules of the process committee at the Center for Genomic Medicine, The Institute of Physical and Chemical Research (RIKEN).

A total of 209 AD patients were recruited from a dermatological clinic at Kaohsiung Medical University hospital and National Taiwan University hospital in Taiwan from January 1st to December 15th in 2008. A diagnosis of AD patients was also based on criteria proposed by Hanifin and Rajka [21]. Information of these participants is provided in Table S2. We took a medical history and performed physical examinations and blood biochemistry exams to exclude other diseases causing pruritus, such as contact dermatitis, asteatotic dermatitis, and metabolic diseases. Suspected cases were biopsied to exclude other mimicking cutaneous diseases, including cutaneous T cell lymphoma. Patients were excluded if they had received any topical treatments for at least 2 weeks or systemic treatment for 2 months prior to the study. Patients were excluded if they had active skin diseases other than AD, including HIV infection and cancers of any origin. Patients visiting the same hospital in the Department of Preventive Medicine for medical diseases other than atopic diseases were referred to the Department of Dermatology were hospital-based controls. They were excluded if they had active skin diseases, past history of AD, allergic asthma, allergic rhinitis, allergic conjuntivitis, cancers of any origin, or HIV infection or were taking oral corticosteroids. A board-certified dermatologist took a medical history, examined the whole surface the body, and assigned a SCORAD severity index score for each subject. Venous peripheral blood was drawn and the serum was stored at −70°C until assayed. IgE levels from AD patients were measured in a College of American Pathologists (CAP) accredited laboratory in the same hospital. The study was approved by the Institutional Review Board of the Hospital. All clinical assessments and specimen collections were conducted according to Declaration of Helsinki principles. Each participant signed an informed written consent form before entering the study. Patients or controls who did not sign the inform consent were excluded.

### Selection of human *ORAI1* polymorphisms for genotyping

Genomic DNA was prepared from peripheral blood samples using standard protocols. To identify SNPs in the human *ORAI1* gene, we sequenced all exons, including a minimum of 200 bases of the flanking intronic sequence, 2 kb of the 5′ flanking region, and a 2 kb continuous 3′ flanking region of the last exon except for regions with interspersed repeats from 24 subjects as described in Japanese population. Pairwise LD was calculated as D′/LOD and r^2^ and the Tag SNPs were selected among 10 SNPs with a frequency of greater than 10% by using the Haploview 4.2 program (http://www.broad.mit.edu/mpg/haploview/). Genotyping of SNPs was performed by the TaqMan allele-specific amplification (TaqMan-ASA) method (Applied Biosystems, Foster City, CA).

In Taiwanese population, we sequenced all exons of *ORAI1* gene in 10 subjects, however, none of SNP was found. We further choose five *ORAI1* tagging SNPs (rs12320939, rs6486795, rs3741595, rs3825175 and rs712853) with a minor allele frequency >10% in the Han Chinese population were selected from the HapMap database (http://www.hapmap.org). Genotyping was performed using the TaqMan Allelic Discrimination Assay (Applied Biosystems, Foster city, CA, USA). The polymerase chain reaction (PCR) was carried out using the ABI7900 Thermal Cycler. After PCR, fluorescence from reaction products was measured and analyzed using the System SDS software version 1.2.3.

### Real-time quantitative RT-PCR

Total RNA from normal human tissues was purchased from Clontech (Mountain View, CA). Each RNA was reverse transcribed with Superscript III reverse transcriptase and oligo dT primers (Invitrogen, Carlsbad, CA). The expression of *ORAI1* transcripts was determined by real-time quantitative reverse transcription polymerase chain reaction (RT-PCR) using SYBR Premix Ex Taq (Takara, Shiga, Japan) with specific primers (5′- ACCTCGGCTCTGCTCTCC -3′ and 5′- GATCATGAGCGCAAACAGG -3′). In all experiments, the amounts of cDNA were standardized by quantification of the housekeeping gene glyceraldehyde-3-phosphate dehydrogenase (*GAPDH*).

### Statistical analysis

We tested agreement with Hardy-Weinberg Equilibrium using a χ^2^ goodness-of-fit test at each locus in both populations. In Japanese population, we then compared differences in genotype frequencies of the polymorphisms between case and control subjects by the Cochran-Armitage trend test, and calculated odds ratios (ORs) with 95 percent confidence intervals (95% CI). We applied Bonferroni correction; the multiplication of the *P* values by four, the number of tag SNPs. In the association study, *P* values of less than 0.05 were considered to be statistically significant. Haplotype frequencies for three loci were estimated, and haplotype association tests were performed using Haploview 4.2. Total IgE levels and expression levels of *ORAI1* transcripts between genotypic groups were tested with the Jonckheere-Terpstra test.Chi-square test was then used to compare differences in allele frequencies and genotype distribution of the polymorphisms between AD and controls.

## Results

### Association of *ORAI1* SNPs with susceptibility to atopic dermatitis

**Figure 1:**
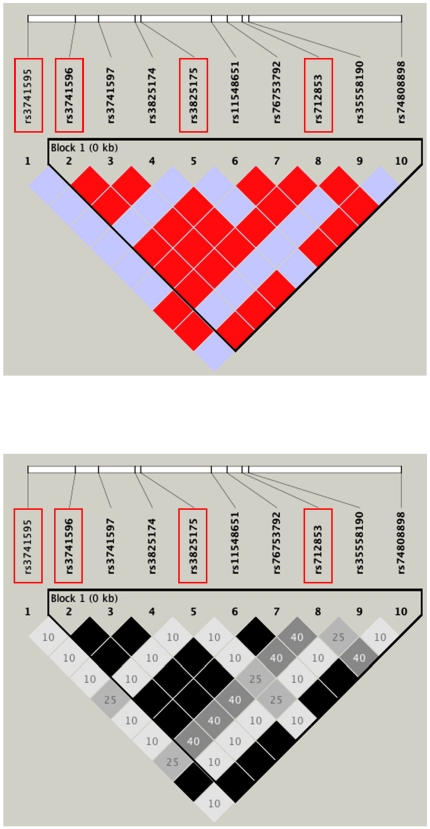
SNPs and pairwise LD map of the *ORAI1* gene. Four boxed polymorphisms were genotyped in the Japanese population. Pairwise D′/LOD (upper) and r^2^ (lower) for all combinations of SNP pairs are shown.

**Figure 2:**
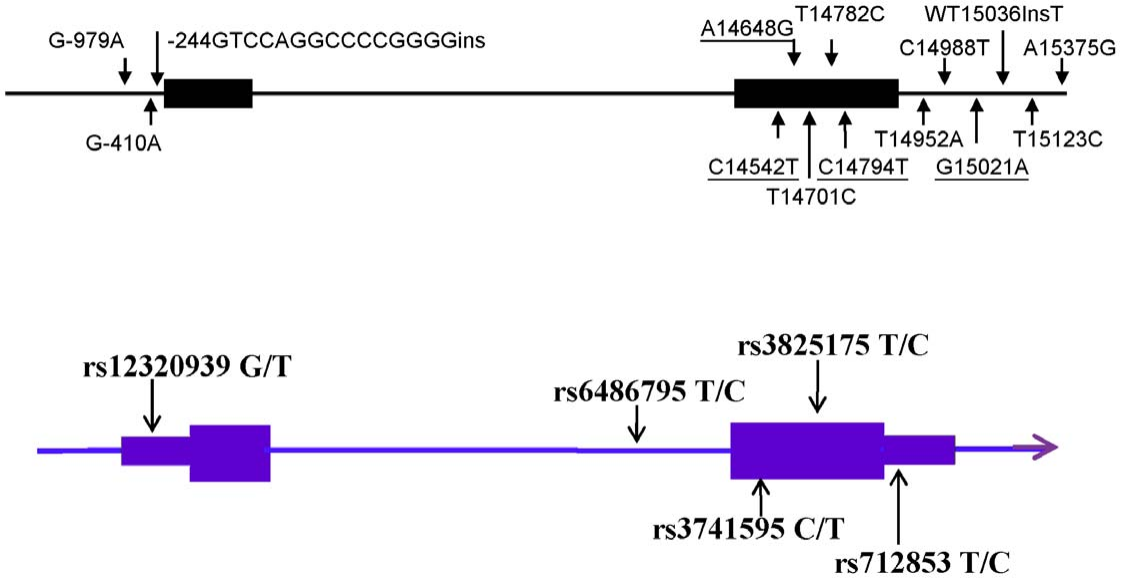
A graphical overview of *ORAI1* gene polymorphisms identified in in the Japanese population (upper). A graphical overview of tSNPs of *ORAI1* gene selected for genotyping in the Taiwanese population.

After extensive examination of *ORAI1* by direct sequencing, we identified 14 polymorphisms (three SNPs in promoter region and five SNPs within the transcript) (Table 1). Eight polymorphisms were contained in the available public databases, NCBI dbSNP (http://www.ncbi.nlm.nih.gov/SNP/). We calculated pairwise linkage disequilibrium (LD) as r^2^ and selected four tag SNPs with a minor allele frequency (MAF) of >10% using the Haploview 4.2 program (http://www.broad.mit.edu/mpg/haploview/). The four tag SNPs captured 10 of the 10 alleles with a mean r^2^ of 0.98 (r^2^>0.76) (Figure 1 and Table S3). The locations of these four SNPs are shown in Figure 2 (upper). In Taiwanese population, five *ORAI1* tagging SNPs with a minor allele frequency >10% in the Han Chinese population were selected from the HapMap database (http://www.hapmap.org). A graphical overview of the genotyped polymorphism is shown in Figure 2 (Lower).

**Table 1:** Locations and allele frequencies of polymorphisms in *ORAI1*.

	SNP^a^	Location	Amino acid	Allele frequency(%)	NCBI^b^
Marker 1	−979G/A	5′ flanking region		2	
Marker 2	−410G/A	5′ flanking region		2	rs116376569
Marker 3	−244GTCCAGGCCCCGGGG×1/×2	5′ flanking region		4	
Marker 4^c^	14542C/T	exon2	I182I	33	rs3741595
Marker 5^c^	14648A/G	exon2	S218G	17	rs3741596
Marker 6	14701T/C	exon2	A235A	17	rs3741597
Marker 7	14782T/C	exon2	V262V	17	rs3825174
Marker 8^c^	14794C/T	exon2	T266T	33	rs3825175
Marker 9	14952T/A	3′ UTR		17	rs11548651
Marker 10	14988C/T	3′ UTR		17	rs76753792
Marker 11^c^	1502A/G	3′ UTR		33	rs712853
Marker 12	15036insT	3′ UTR		33	rs35558190
Marker 13	15123T/C	3′ UTR		2	
Marker 14	15375A/G	3′ flanking region		17	rs74808898

a Numbering according to the genomic sequence of *ORAI1* (AC140062.11).

Position 1 is the A of the initiation codon.

b NCBI, Number from the dbSNP of NCBI (http://www.ncbi.nlm.nih.gov/SNP/).

c SNPs were genotyped in both Taiwanese and Japanese population.

We next carried out case-control association studies of the four and five SNPs in the 1540 Japanese subjects and 938 Taiwanese subjects. The control genotypes did not deviate from Hardy-Weinberg equilibrium. As shown in Table 2, a nonsynonymous *ORAI1* SNP (rs3741596, Ser218Gly) showed significant associations with susceptibility to atopic dermatitis (*P* = 0.002, OR = 1.36, 95% CI 1.12–1.64) by the Cochran-Armitage trend test in Japanese population. Other variants did not show a significant association after Bonferroni correction. In Taiwanese population, rs6486795 and rs3741595 were significantly associated with susceptibility to atopic dermatitis under the dominant and allelic models.

**Table 2:** Genotype counts for *ORAI1* and atopic dermatitis susceptibility in Japanese and Taiwanese population.

			Japanese (case-control association analysis)						Taiwanese (case-control association analysis)					
			Genotype^a^			Allele*2			Genogype			Allele*2		
db SNP ID	Allele 1/2	Subjects	1/1	1/2	2/2	Frequency of allele 2	*P* a	OR (95% c.i.)	1/1	1/2	2/2	Frequency of allele 2	*P*	OR (95% c.i.)
rs12320939	G/T	AD							50	90	67	0.54	0.050	1.25 (1.00–1.55)
		Control							184	341	165	0.49		
rs6486795	T/C	AD							59	93	47	0.47	0.0004	1.50 (1.20–1.88)
		Control							277	320	98	0.37		
rs3741595	C/T	AD	323	160	25	0.21	0.275	0.90 (0.75–1.08)	83	93	31	0.37	0.0001	1.59 (1.27–2.01)
		Control	622	343	58	0.22			370	275	53	0.27		
rs3741596	A/G	AD	315	177	19	0.21	0.002	1.36 (1.12–1.64)						
		Control	717	283	27	0.16								
rs3825175	C/T	AD	197	243	71	0.38	0.245	0.91 (0.78–1.06)	89	81	37	0.37	0.187	0.86 (0.69–1.08)
		Control	379	476	171	0.40			239	346	114	0.41		
rs712853	G/A	AD	165	266	80	0.42	0.027	1.19 (1.02–1.38)	26	71	109	0.70	0.035	1.29 (1.02–1.64)
		Control	397	479	144	0.38			97	303	301	0.65		

a *P* values represent the Cochran-Armitage trend *P* for case-control comparisons.

### Haplotypes of *ORAI1* and their association with the disease occurrence of atopic dermatitis

**Figure 3:**
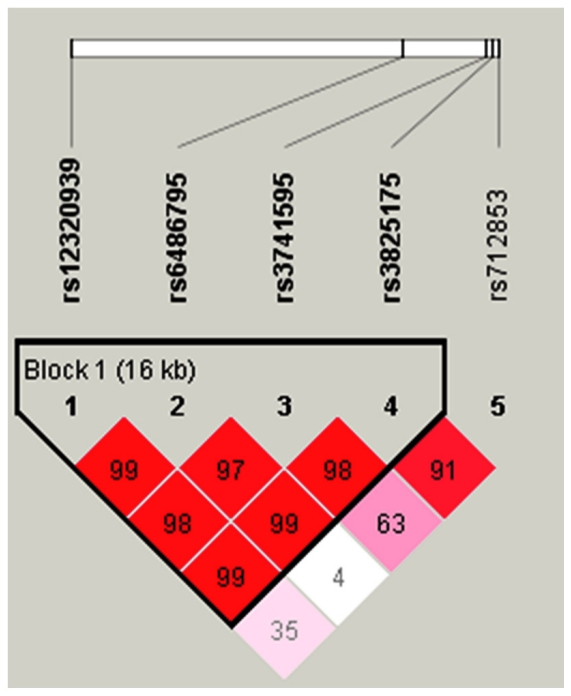
*ORAI1* gene LD and haplotype block structure in AD. The number on the cell is the LOD score of D′.

To further identify the effects of Haplotypes of *ORAI1* to atopic dermatitis, we constructed the haplotypes of the three SNPs to estimate the frequency of each haplotype in controls in the Japanese population (Table 3). Three common haplotypes were identified in the Japanese population. Haplotype C-G-C-A (rs3741595, rs3741596, rs3825175, and rs712853) of *ORAI1* was significantly associated with atopic dermatitis. A *P* value of 0.0017 was obtained by using the Haploview 4.2 program. In the Taiwanese population, the haplotype block structure of *ORAI1* was shown in Figure 3. Among five haplotypes, subjects with T-C-T-C haplotype (rs12320939, rs6486795, rs3741595 and rs3825175) have a significant risk to AD (OR = 1.54, 95% CI = 1.18–2.01, P = 0.0014) (Table 4).

**Table 3:** Haplotype frequency of four SNPs in *ORAI1* in Japanese population.

					n		Frequency			Odds ratio
	rs3741595	rs3741596	rs3825175	rs712853	AD	Control	AD	Control	*P* value	(95% c.i.)
Haplotype 1	C	A	T	G	386	819	0.38	0.40		
Haplotype 2	T	A	C	G	212	459	0.21	0.22		
Haplotype 3	C	A	C	A	211	435	0.20	0.21		
Haplotype 4	C	G	C	A	216	337	0.21	0.16	0.0017	1.36 (1.12–1.64)
					1026	2050	1.00	1.00		

**Table 4:** Haplotype frequency of four SNPs in *ORAI1* in Taiwanese population.

					n		Frequency			Odds ratio
	rs12320939	rs6486795	rs3741595	rs3825175	AD	Control	AD	Control	*P* value	(95% c.i.)
Haplotype 1	T	C	T	C	148	370	0.38	0.27	0.0014	1.54 (1.18–2.01)
Haplotype 2	T	C	C	C	36	130	0.09	0.10		
Haplotype 3	T	T	C	C	33	154	0.08	0.11		
Haplotype 4	G	T	C	C	30	142	0.08	0.10		
Haplotype 5	G	T	C	T	143	550	0.36	0.41		
					390	1346	0.99	0.99		

If the frequencies less than 1% were excluded.

### Expression of *ORAI1* mRNA in skin, immune tissues

**Figure 4:**
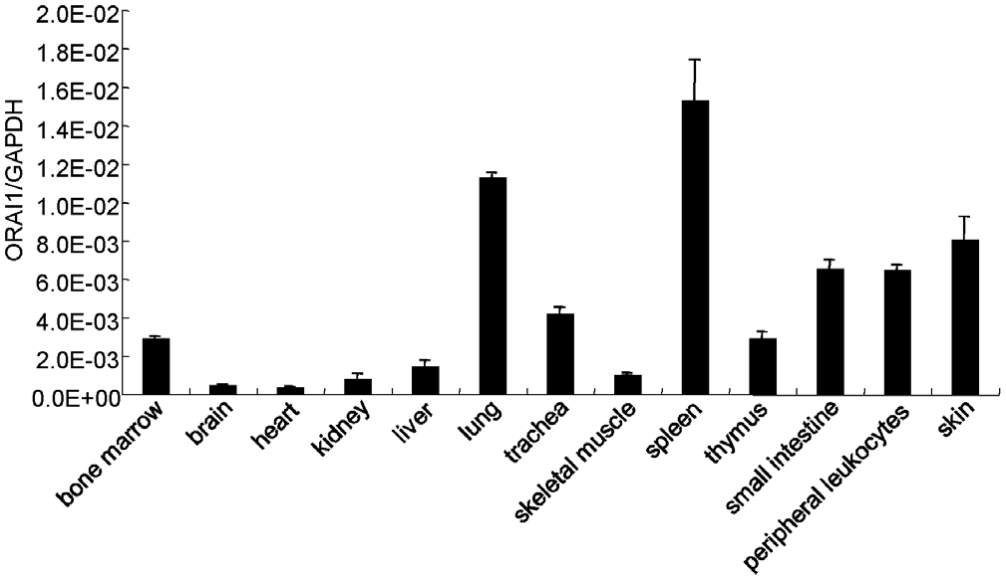
Expression of *ORAI1* mRNA in different tissues. Quantities of total RNA extracted from normal human tissues were determined by real-time quantitative reverse transcription polymerase chain reaction (RT-PCR). The results were normalized to GAPDH transcripts.

To investigate the *ORAI1* mRNA expression in various human tissues, we conducted real-time quantitative RT-PCR. As shown in Figure 4, *ORAI1* mRNA was expressed abundantly in all tissues examined, especially in the tissues of lung, spleen, and skin.

### rs3741597 is associated with the expression level of the *ORAI1* transcript

**Figure 5:**
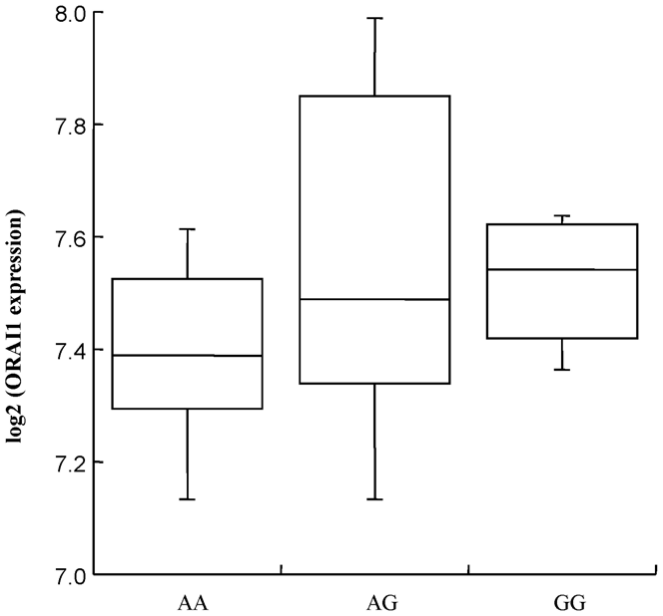
Differential expression of an *ORAI1* transcript (NM_032790) of EBV-transformed lymphoblastoid cell lines from HapMap-JPT (Japanese in Tokyo) subjects for each rs3741597 genotype. *P* value was calculated by the Jonckheere-Terpstra test.

We next assessed whether the rs3741597 genotype correlated with the mRNA levels of *ORAI1* transcript (NM_032790). The *ORAI1* mRNA expression data for the EBV-transformed lymphoblastoid cell lines from 42 JPT HapMap subjects were analyzed by the Jonckheere-Terpstra trend test. As shown in Figure 5, the expression level of transcripts of NM_032790 was positively correlated with the rs3741597 genotype (*P* = 0.040). These results suggested that rs3741597 or other SNPs, 14648A/G (rs3741596), 14782T/C (rs3825174), 14952T/A (rs11548651), 14988C/T (rs76753792), and 15375A/G (rs74808898), in strong linkage disequilibrium with rs3741597 might influence susceptibility to atopic dermatitis through higher expression of an *ORAI1* transcript.

## Discussion

We screened the polymorphisms of *ORAI1* and performed a case-control association study and a haplotype analysis. A total of 14 genetic variants was identified from Japanese population, 3 SNPs are novel. Our results showed a significant association between AD and a non-synonymous SNP (rs3741596, Ser218Gly) in the human *ORAI1* gene in the Japanese population. rs3741596 was genotyped in the Taiwanese population, however, the MAFs of this SNP is less than 1% which is different from the allele frequency of Japanese population (data not shown). In addition, rs3741595 of *ORAI1* is associated with the risk of AD in the Taiwanese population but not in the Japanese population. We also performed I^2^ index to assess heterogeneity on three SNPs which were genotyped in both Taiwan and Japan. The I^2^ indexes of rs3825175 and rs712853 showed no heterogeneity on these locus. The heterogeneity only occurred on rs3741595 (I^2^ Index = 93%) We attribute this to the different genetic backgrounds in the populations, due to variation in allele frequencies, population admixture, heterogeneity of the phenotype between populations.

The rs3741597 genotype correlated with the mRNA levels of the *ORAI1* transcript. Another three SNPs (14952T/A, 14988C/T, and 15375A/G) located in the 3′ flanking region, which are in strong LD with rs3741597, may influence higher expression of an *ORAI1* transcript. MicroRNAs (miRNA) are small non-coding RNAs that control gene expression by preferentially binding to the 3′-untranslated regions (3′-UTR) of the target genes [22]. Therefore, further functional analysis of the 3′-UTR of the *ORAI1* gene should be conducted in order to clarify the mechanism underlying this susceptibility.

Several diseases have been linked to the *ORAI1*-mediated calcium influx. Feske et al. identified a mutation in the Orai1 from SCID patients. This missense mutation caused a reduction in the SOC influx and a decrease in immune response [19]. Studies from the Orai1-knockout mice have shown an important role of this gene in the activation of inflammatory reactions in mast cells [20], [23]. To study the role of *ORAI1*, we analyzed the tissue distribution of ORAI1. The highest expression of ORAI1 was found in the spleen, an organ involved in immune function. The tissue distribution of ORAI1 in our study revealed the potential importance of ORAI1 in the regulation of immune system—a finding consistent with that reported previously. In addition, the expression of ORAI1 was found to be higher in the proliferative vascular smooth muscle cells [24]. Furthermore, results obtained from ORAI1 knockdown cells indicated a reduction of cell proliferation in both endothelial and breast cancer cells [25], [26]. Studies using keratinocytes have shown that calcium is an important regulator of cell proliferation and differentiation [27]. Dysregulated apoptosis in keratinocytes contributes to the progress of AD [28]. Hence, genetic polymorphisms may result in the changes of gene expression level of *ORAI1* that further contribute to the dysregulated growth in keratinocytes, leading to a defective skin-barrier formation. Cell-based physiological studies in the keratinocytes are required to identify the role of *ORAI1* in AD.

STIM1 and ORAI1 are the two major components in the regulation of calcium entry through store-operated calcium channels. Co-expression of STIM1 and Orai1/CRACM1 results in the amplification of store-operated Ca^2+^ influx signals [1], [29], [30]. The SOC entry pathway is influenced by ORAI1 or STIM1 knockdown [31], [32]. Importantly, the overexpression of orai1 also causes the attenuation of store-operated Ca^2+^ influx [33]. Two possible mechanisms were proposed. The oligomers formed by the overexpressed ORAI1 may lose sensitivity to the signals released from calcium store [34]. Soboloff et al. suggested that the coupling stoichiometry between Orai1 and STIM1 is not unity, therefore, overexpression of Orai1 may influence the functional compositions of SOC [30]. Combined the findings from other groups [34], our results propose that the genetic polymorphisms in the 3′-UTR of the *ORAI1* gene may change the expression level of Orai1, which, in turn, may cause dysfunction of calcium channels and immune responses. However, the coupling stoichiometry between the different expression levels of ORAI1 should also be further considered.

Previous studies have revealed the significant association between genetic polymorphisms of *ORAI1* and inflammatory diseases such as ankylosing spondylitis and calcium nephrolithiasis [35], [36]. In this study, we identified 14 genetic variants including 3 novel SNPs in the *ORAI1* gene. In a total of 2,478 subjects (938 Taiwanese and 1540 Japanese), our results indicated that different genetic polymorphisms of *ORAI1* are associated with AD susceptibility in the Japanese, and Taiwanese populations. This is the first report to state the relationship between the genetic polymorphisms of *ORAI1* and allergic diseases. Given the polygenic nature of allergic diseases such as AD, the susceptibility gene *ORAI1* could provide a new clue in the pathogenesis of AD. The prevalence of atopic dermatitis in Taiwan is around 6.7%, therefore, this study should reach a power level of 0.97 (case 209; control 729). Although further replication studies in larger Taiwanese population is needed, it is likely to variants in the ORAI1 gene play a role in susceptibility to AD in both Japanese and Taiwanese populations. Further study on the relationship between the genotype of *ORAI1* and the downstream functional relevance during dermal inflammation should be conducted in order to understand the etiology of AD.

## Supporting Information

Table S1 **Basal characteristics of patients with Atopic Dermatitis (AD) and of normal controls in Japanese population.**
(DOC)

Table S2 **Basal characteristics of patients with Atopic Dermatitis (AD) and of normal controls in Taiwanese population.**
(DOC)

Table S3 **Pairwise linkage disequilibrium for all possible two-way comparisons among 10 polymorphisms in** ***ORAI1*** **with 24 Japanese volunteers.**
(DOC)
