# Supplementary material for: ORAI1 Genetic Polymorphisms Associated with the Susceptibility of Atopic Dermatitis in Japanese and Taiwanese Populations
Source: PLoS One. 2012 Jan 13;7(1):e29387. doi: 10.1371/journal.pone.0029387 (PMC3258251; doi:10.1371/journal.pone.0029387)
Supplement: Table S1 — Basal characteristics of patients with Atopic Dermatitis (AD) and of normal controls in Japanese population. (DOC) [file pone.0029387.s001.doc]

**Supporting tables**

| **Tables S1.** Basal characteristics of patients with Atopic Dermatitis (AD) and of normal controls in Japanese population | | |
| --- | --- | --- |
| Characteristics | Patients with AD | Normal control |
| Number of subjects | 513 | 1027 |
| Age (year) mean ± SD | 29.0 ± 9.0 | 48.5 ± 13.7 |
| Male (%) | 52.0 | - |
|  | | |
