# Supplementary material for: ORAI1 Genetic Polymorphisms Associated with the Susceptibility of Atopic Dermatitis in Japanese and Taiwanese Populations
Source: PLoS One. 2012 Jan 13;7(1):e29387. doi: 10.1371/journal.pone.0029387 (PMC3258251; doi:10.1371/journal.pone.0029387)
Supplement: Table S2 — Basal characteristics of patients with Atopic Dermatitis (AD) and of normal controls in Taiwanese population. (DOC) [file pone.0029387.s002.doc]

| **Tables S2.** Basal characteristics of patients with Atopic Dermatitis (AD) and of normal controls in Taiwanese population | | |
| --- | --- | --- |
| Characteristics | Patients with AD | Normal control |
| Number of subjects | 209 | 729 |
| Age (year) mean ± SD | 56.0 ± 21.2 | 42.5± 19.3 |
| Male (%) | 64.9 | 53.0 |
|  | | |
