# Supplementary material for: ORAI1 Genetic Polymorphisms Associated with the Susceptibility of Atopic Dermatitis in Japanese and Taiwanese Populations
Source: PLoS One. 2012 Jan 13;7(1):e29387. doi: 10.1371/journal.pone.0029387 (PMC3258251; doi:10.1371/journal.pone.0029387)
Supplement: Table S3 — Pairwise linkage disequilibrium for all possible two-way comparisons among 10 polymorphisms in ORAI1 with 24 Japanese volunteers. (DOC) [file pone.0029387.s003.doc]

**Tables S3.** Pairwise linkage disequilibrium for all possible two-way comparisons among 10 polymorphisms in *ORAI1* with 24 Japanese volunteers

| D’/LOD | Marker4 | Marker5 | Marker6 | Marker7 | Marker8 | Marker9 | Marker10 | Marker11 | Marker12 | Marker14 |
| --- | --- | --- | --- | --- | --- | --- | --- | --- | --- | --- |
| r^2^ | 14542C/T | 14648A/G | 14701T/C | 14782T/C | 14794C/T | 14952T/A | 14988C/T | 15021G/A | 15036insT | 15375A/G |
| Marker 4^a^ |  | 1.00 | 1.00 | 1.00 | 1.00 | 1.00 | 1.00 | 1.00 | 1.00 | 1.00 |
| Marker 5^a^ | 0.10 |  | 1.00 | 1.00 | 1.00 | 1.00 | 1.00 | 1.00 | 1.00 | 1.00 |
| Marker6 | 0.10 | 1.00 |  | 1.00 | 1.00 | 1.00 | 1.00 | 1.00 | 1.00 | 1.00 |
| Marker7 | 0.10 | 1.00 | 1.00 |  | 1.00 | 1.00 | 1.00 | 1.00 | 1.00 | 1.00 |
| Marker 8^a^ | 0.25 | 0.10 | 0.10 | 0.10 |  | 1.00 | 1.00 | 1.00 | 1.00 | 1.00 |
| Marker9 | 0.10 | 1.00 | 1.00 | 1.00 | 0.10 |  | 1.00 | 1.00 | 1.00 | 1.00 |
| Marker10 | 0.10 | 1.00 | 1.00 | 1.00 | 0.10 | 1.00 |  | 1.00 | 1.00 | 1.00 |
| Marker 11^a^ | 0.25 | 0.40 | 0.40 | 0.40 | 0.25 | 0.40 | 0.40 |  | 1.00 | 1.00 |
| Marker12 | 1.00 | 0.10 | 0.10 | 0.10 | 0.25 | 0.10 | 0.10 | 0.25 |  | 1.00 |
| Marker14 | 0.10 | 1.00 | 1.00 | 1.00 | 0.10 | 1.00 | 1.00 | 0.40 | 0.10 |  |

^a^SNPs genotyped in this study.
